# A novel genomic inversion in Wiskott-Aldrich–associated autoinflammation

**DOI:** 10.1016/j.jaci.2016.03.007

**Published:** 2016-08

**Authors:** Immacolata Brigida, Samantha Scaramuzza, Dejan Lazarevic, Davide Cittaro, Francesca Ferrua, Lorena Leonardelli, Maria Alessio, Ornella Forma, Chiara Lanzani, Gianluca Viarengo, Fabio Ciceri, Momcilo Jankovic, Fernando Pesce, Alessandro Aiuti, Maria Pia Cicalese

**Affiliations:** a San Raffaele Telethon Institute for Gene Therapy (SR-TIGET), IRCCS San Raffaele Scientific Institute, Milan, Italy; b Center for Translational Genomics and BioInformatics-Hospital San Raffaele, IRCCS San Raffaele Scientific Institute, Milan, Italy; c Pediatric Immunohematology and Bone Marrow Transplantation Unit, IRCCS San Raffaele Scientific Institute, Milan, Italy; d Vita-Salute San Raffaele University, Milan, Italy; e Rheumatology Unit, Department of Pediatrics, Federico II University of Naples, Naples, Italy; f Vulnology Nursing Service, IRCCS San Raffaele Scientific Institute, Milan, Italy; g Nephrology Department, IRCCS San Raffaele Scientific Institute, Milan, Italy; h Immunohaematology and Transfusion Service, Fondazione IRCCS Policlinico S. Matteo, Pavia, Italy; i Haematology and Bone Marrow Transplantation Unit, IRCCS San Raffaele Scientific Institute, Milan, Italy; j University of Milano-Bicocca, Fondazione Monza e Brianza per il Bambino e la sua Mamma, San Gerardo Hospital, Monza, Italy; k A.S.O., “SS Antonio e Biagio e Cesare Arrigo,” Alessandria, Italy

To the Editor:

Wiskott-Aldrich syndrome (WAS) is an X-linked disorder characterized by thrombocytopenia, eczema, and immunodeficiency. Up to 70% of patients with WAS present with at least 1 autoimmune or autoinflammatory episode, and many of them suffer from recurrent or multiple events.^1^, ^2^, ^3^ IL-1 new-generation blockers have been used in patients exhibiting clinical symptoms compatible with an autoinflammatory condition,^4^ but have not been reported in WAS. Here, we describe a patient with WAS with a peculiar large genomic inversion presenting with multiple manifestations of immune dysregulation, in whom autoinflammatory manifestations improved after the use of anakinra (IL-1 receptor antagonist, Kineret).

A 11.6-year-old boy was referred to our center for suspected immunodeficiency. The patient presented with a history of microthrombocytopenia since birth and eczema in the first years of life, suggestive of WAS. Analysis of WAS protein (WASp) expression was reported abnormal, but Sanger sequencing on DNA did not reveal mutations. From 1.5 years of age he underwent recurrent episodes of postinfectious vasculitis of the lower limbs and arthritis. At 7.5 years, he presented with a bilateral pneumonia that triggered Schonlein-Henoch purpura with fever and arthritis, managed with oral steroids. Subsequently, a nephritic-nephrotic syndrome was treated with antihypertensive treatment and high-dose corticosteroids (CCS), with partial response. Cyclosporin A (CyA) and CCS led to remission of renal disease, which relapsed after CyA was stopped. Intravenous high-dose CCS and anti-CD20 mAb did not lead to substantial improvement. CyA and low-dose prednisone were restarted with partial benefit. However, the patient experienced varicella zoster reactivation on his half-right-face, with sequelae to the right eye (anterior and posterior uveitis with acute retinitis) requiring a vitrectomy, and severe impairment of visual function. An anterior uveitis at the left eye was treated with steroids. At the age of 9.8 years, he developed clinical and histological features of pancolitic Crohn disease, managed with an increase in CCS, as well as arthritis and histologically confirmed vasculitis and eventually pyoderma gangrenosum (PG) on the hips, buttocks, and upper and lower limbs. Crohn disease was not responsive to infliximab, thalidomide, cyclophosphamide, or high-dose intravenous steroids, while adalimumab (Humira) resulted in an initial benefit (see Table E1 in this article's Online Repository at www.jacionline.org). The patient presented with fistulas and perianal abscesses when he was 10.7 years old and he underwent several fistulectomies and removal of granulation tissue in the perianal area by “cone-like technique.” For the poor control of the enterocolitis, a subtotal colectomy with terminal ileostomy was performed at age 11 years.

When the patient was referred to our center, he was on adalimumab and low-dose CCS with a good control of bowel disease, but still showed severe manifestations of PG on the upper limbs and in the perianal area (Fig 1, *A*; see Table E2 in this article's Online Repository at www.jacionline.org). His parents signed informed consent for research investigations (protocol Tiget06).

Because of the strong suspicion of WAS, whole-genome sequencing was performed and an inversion of 6kb spanning from the promoter to the intronic region between exons 7 and 8 was detected (see Fig E1 in this article's Online Repository at www.jacionline.org). Specific primers in this region identified the precise breaking points (see Tables E3 and E4 in this article's Online Repository at www.jacionline.org; Fig 2, *B*). The rearranged allele was present in the patient and his mother, whereas the patient's aunt was unaffected (data not shown and Fig 2, *A*-*C*).

RNA analyses showed an aberrant transcript produced from the inverted region (Fig 2, *D*). WASp expression, analyzed by flow cytometry (see Fig E2, *A*, in this article's Online Repository at www.jacionline.org), was deeply reduced in peripheral blood T-, B-, and natural killer lymphocytes and monocytes (data not shown) while it was undetectable by Western blot performed with an antibody recognizing the N-terminal portion of WASp including exons 7 and 8 (Fig E2, *B*). WASp expression was restored in the patient's T-cell line transduced with a lentiviral vector expressing WASp under the control of the autologous 1.6-kb long promoter^5^ (Fig E2, *C*).

The start of low-dose methotrexate (Reumaflex) and the increase in prednisone led to a moderate improvement in the PG after 3 months (Fig 1, *B*), but shortly after the patient underwent a reactivation of vasculitis and arthritis with systemic inflammation that was not controlled by multiple immunosuppressive and anti-inflammatory drugs.

On the basis of the reported efficacy of IL-1 blockers in the treatment of autoinflammatory manifestations and of PG,^4^ anakinra was started as an off-label drug titrating the dose from 1 up to 3 mg/kg/day. This led to a resolution of vasculitis and arthritis and to a decrease in the inflammation indexes within few days (Fig 1, *D*) with dramatic improvement in the PG skin lesions during the following 5 months (Fig 1, *C*).

The patient was enrolled in a gene therapy trial based on autologous gene-corrected hematopoietic stem cells (clinicaltrials.gov #NCT01515462), mobilized with G-CSF and plerixafor. Treatment with anakinra was discontinued 48 hours before mobilization, but was soon restarted because of the increase in white blood cells and inflammation indexes with exacerbation of skin lesions, arthralgia, and hematuria, and led again to a rapid laboratory and clinical remission (data not shown). Notably, the use of anakinra allowed a successful mobilization with G-CSF without the occurrence of other autoinflammatory manifestations. To our knowledge, this is the first reported case of use of IL-1R blocker in a patient with WAS, with clinical benefit.

This case is very emblematic for several reasons. Whole-genome sequencing complemented by specific breakpoint sequencing allowed the identification of the inversion with intact exon sequences, elucidating the previous normal genetic analysis. Complex genomic rearrangements involving inversions are generally noncanonical gene conversion events^6^ and could have occurred in an ancestor allele in the family through a *de novo* mutation occurring in the mother.

Autoimmune and autoinflammatory manifestations in patients with WAS typically present early in life, are often refractory to therapy, and are associated with a worse clinical prognosis and an increased risk of developing a malignancy.^3^, ^7^ Our patient's autoinflammatory manifestations were resistant to several immunosuppressive drugs and the use of CyA was associated with a severe viral complication. Anakinra dramatically improved PG, vasculitis, and arthritis, showed a good safety profile, and allowed stabilization of the patient for definitive treatment. The response to anakinra suggests that the dysregulation of the innate immune system is involved in the genesis of autoinflammatory manifestations in patients with WAS and shows that IL-1 may serve in selected cases as a target for therapy, avoiding the use of other classes of immunosuppressors that can increase the risk for severe infections.

It has been hypothesized that defects in chemotaxis and podosomes formation in WASp-deficient cells may favor the onset of autoinflammatory manifestations. In addition, a recent study in a patient with aggressive PG showed a critical role for proline-serine-threonine phosphatase interacting protein 1, which is involved in cytoskeletal regulatory functions through interaction with WASp, in the Pyogenic Arthritis, Pyoderma gangrenosum, and Acne syndrome.^8^ A greater understanding of the role of WASp in inflammation and of potential pathways that may be targeted therapeutically to modulate immunity in WAS is desirable to improve the management of the affected patients while waiting for definitive treatment by stem cell transplantation or gene therapy.

## Figures and Tables

**Fig E1:**
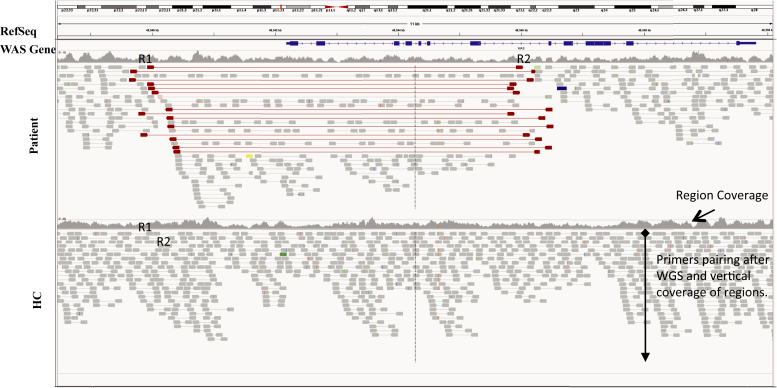
Graphical representation of WGS results. *WAS* gene and coverage are indicated. Primers R1 and R2 for Illumina sequencing that pair correctly are represented in gray. The red lines in the patient indicate the pairing in the region spanning the inversion and their specific orientation. *HC*, Healthy control; *WGS*, whole-genome sequencing.

**Fig E2:**
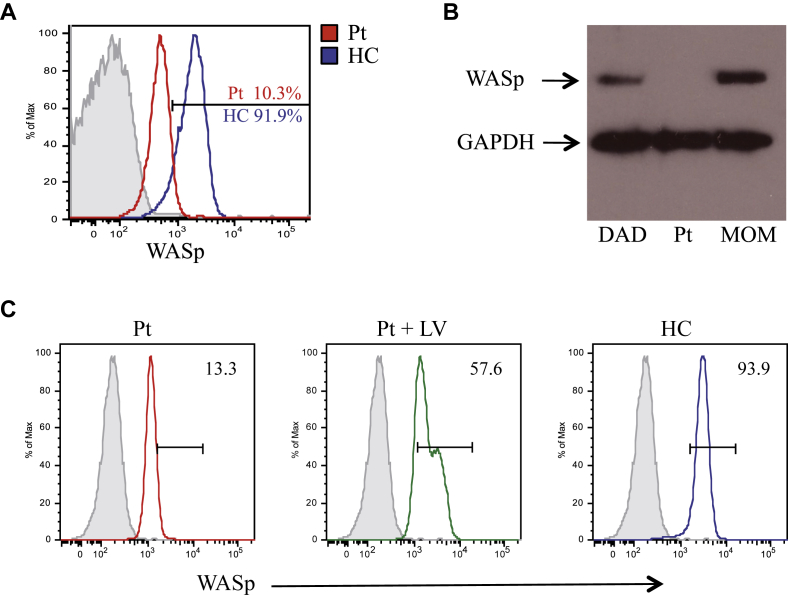
WASp expression. **A,** Flow cytometry characterization of WASp expression in patient and HC lymphocytes. Percentage of WASp+ cells is reported on histograms. Detection of WASp was performed after permeabilization (Cytofix/Cytoperm kit; BD Biosciences, San Jose, Calif) by a noncommercial rabbit polyclonal antibody (“EFOB,” generated against a WASp peptide), kindly provided by GSK. Gray line: negative control. **B,** Western blot performed on untransformed T-cell line generated *in vitro* from patient and his parents. Antibody for detection: polyclonal H250 (BD). **C,** Restoration of WASp expression in a patient's T-cell line after transduction with LV. *GAPDH*, Glyceraldehyde 3-phosphate dehydrogenase; *HC*, healthy control; *LV*, lentiviral vector; *Pt*, patient.

**Table E1:** Summary of treatment of possible autoimmune and autoinflammatory manifestations

Treatment	Response
Nephritic/nephrotic syndrome	
Steroids	Partial
Steroids + CyA	Remission, then relapse when CyA stopped
Steroids + anti-CD20 mAb	No
Steroids + CyA	Partial; infectious complications
Crohn disease	
Steroids + CyA	No
Steroids + CyA + IVIG	No
Steroids + CyA + infliximab	Partial, then relapse
Steroids + CyA + talidomide	No
Steroids + cyclophosphamide	No
Steroids + adalimumab	Yes
PG	
Steroids + dapsone + adalimumab	No
Steroids + dapsone + adalimumab + MTX	Partial
Steroids + dapsone + anakinra	Yes
Arthritis and vasculitis	
Steroids + CyA	Remission, then relapse
Steroids + CyA + infliximab	No
Steroids + dapsone + adalimumab + MTX	No
Steroids + dapsone + anakinra	Yes

*IVIG*, Intravenous immunoglobulin; *MTX*, methotrexate.

**Table E2:** Clinical data and significant laboratory findings at WAS diagnosis

Age of onset/diagnosis of disease	Neonatal/11.6 y
Genetic analyses (whole-genome sequencing)	inv(X)g.5721-11840
Clinical manifestations	Microthrombocytopenia Mild eczema, severe infections, arthritis, vasculitis Multiple Henoch-Schonlein purpura episodes Nephritic-nephrotic syndrome, panuveitis Steroid-resistant Chron enterocolitis Perianal fistulas and abscesses PG
Zhu score	5A^E1^
WBC (×10^9^/L) (normal value, 4.8-10.8 × 10^9^/L)	14.4
Neutrophils (×10^9^/L) (normal value, 1.8-7.7 × 10^9^/L)	12.7
Lymphocytes (×10^9^/L)	1
Platelets range (×10^9^/L)	20-40
MPV (fl) (normal value, 9.1-12.5)	8
CD3+ (cells/μL) (normal value, 1000-2100)^E2^	498
CD3+CD4+ (cells/μL) (normal value, 500-1300)^E2^	364
CD3+CD8+ (cells/μL) (normal value, 220-950)^E2^	91
CD19+ (cells/μL) (normal value, 180-600)^E2^	101
CD16+CD56+ (cells/μL) (normal value, 170-670)^E2^	338
CD4+CD45RA+ (cells/μL) (normal value, 320-1000)^E3^	152
CD4+CD45RO+ (cells/μL) (normal value, 230-630)^E3^	201
CD8+CD45RA+ (cells/μL) (normal value, 310-900)^E3^	159
CD8+CD45RO+ (cells/μL) (normal value, 70-390)^E3^	40
Vβ repertoire	Polyclonal on CD3+
IgG (g/L) (normal value, 7.07-19.19)^E4^	7.69 (on IVIG supplementation)
IgA (g/L) (normal value, 0.60-2.7)^E4^	6.68
IgM (g/L) (normal value, 0.61-2.76)^E4^	0.33
Autoimmunity screening∗	Negative
Antiplatelets antibody (indirect test)	Positive
LLAC	Negative

*ACA*, Anticardiolipin antibody; *AMA*, antimitochondrial antibody; *ANA*, antinuclear antibody; *ASCA*, anti–*Saccharomyces cerevisiae* antibody; *ASMA*, anti–smooth muscle antibody; *cANCA*, cytoplasmic antineutrophilic cytoplasmic antibodies; *ds-DNA*, double-strand DNA antibody; *ENA*, extractable nuclear antibodies; *IVIG*, intravenous immunoglobulin; *LLAC*, lupus-like anticoagulant; *LKM1*, liver kidney microsomal type 1 antibody; *pANCA*, protoplasmic antineutrophilic cytoplasmic antibodies; *WBC*, white blood cell.

∗ Including Coombs test, ANA, ENA, AMA, ASMA, anti–LKM1 antibody, anti–dS-DNA antibody, ASCA, cANCA, pANCA, ACA, and anti–β2 glicoprotein antibody.

**Table E3:** Primers for the detection of DNA inversion

Primer	Orientation	Sequence	PCR cycles
A	FOR	CTGGAGATACGCCTAGCACA	94°C 3 min, 94°C 30 s, 55°C 30 s, 72°C 1 min × 40 cycles, 72°C 10 min
	REV	TCTGAGGGCTGTAGGGTTTG	
B	FOR	AAGGTGGGCACATGGGTAG	94°C 3 min, 94°C 30 s, 55°C 30 s, 72°C 1 min × 40 cycles, 72°C 10 min
	REV	TCTCAGCAGTGGTCTTTGGA	

**Table E4:** Primers for amplification of cDNA

Region	Orientation	Sequence	PCR cycles
EXON1-4 (279bp)	FOR	CGAAAATGCTTGACGCTGGC	94°C 3 min, 94°C 30 s, 54.6°C 30 s, 72°C 1 min × 40 cycles, 72°C 10 min
	REV	CTCGTCCTCGTCTGCAAAGT	
EXON7-10 (303bp)	FOR	ACCCAGTGGATTCAAGCATGT	94°C 3 min, 94°C 30 s, 54.6°C 30 s, 72°C 1 min × 40 cycles, 72°C 10 min
	REV	GGACCAGAACGACCCTTGTT	
EXON2-7 (449bp)	FOR	GTCCTACTTCATCCGCCTTTAC	94°C 3 min, 94°C 30 s, 55.8°C 30 s, 72°C 1 min × 40 cycles, 72°C 10 min
	REV	TCTTCCCTGAGCGTTTCTTATC	
EXON7-8 (73bp)	FOR	GTGGATTCAAGCATGTCAGCC	94°C 3 min, 94°C 30 s, 55.8°C 30 s, 72°C 1 min × 40 cycles, 72°C 10 min
	REV	TCTGGGTCGAGGTTGTTCAC	

**Fig 1:**
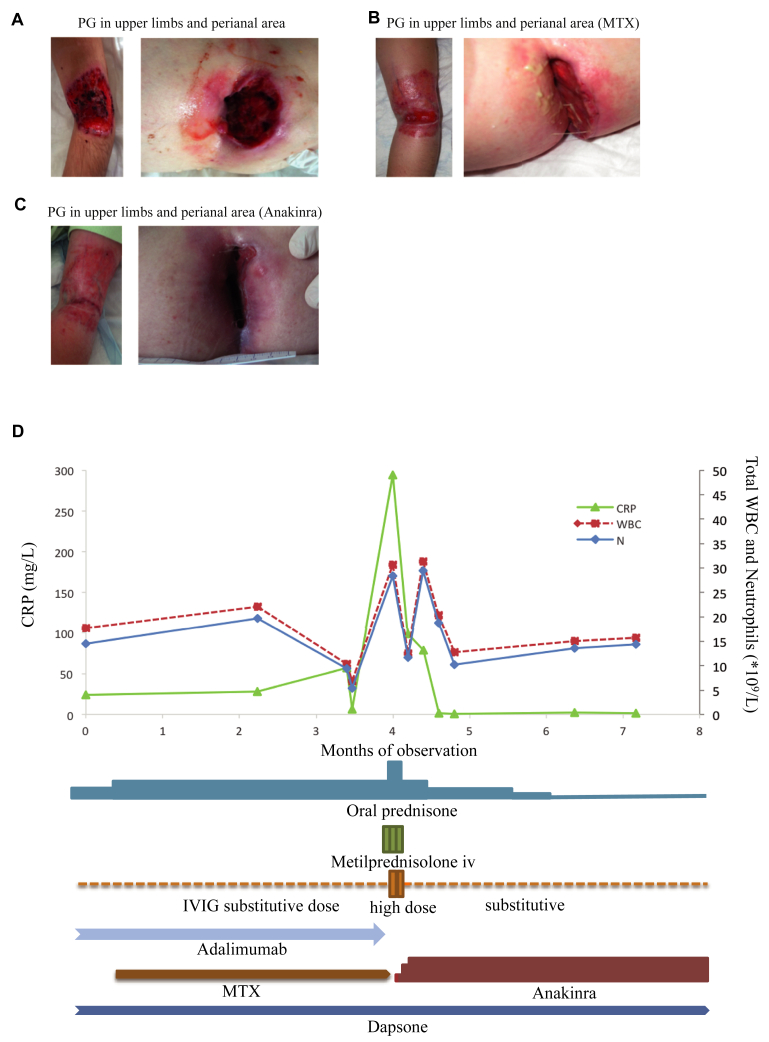
Skin lesions and biochemical markers in a patient with WAS with autoinflammatory manifestations. **A,** Patient at the time of WAS diagnosis. **B,** Patient after 3 months of treatment with MTX. **C,** Patient after 5 months of treatment with anakinra. **D,** Inflammation pattern during several immunosuppressive and antinflammatory treatments administered. Increase in CRP, WBC, and neutrophils (N) at month 4 and amelioration after treatment with anakinra. *CRP*, C-Reactive protein; *IV*, intravenous; *IVIG*, intravenous immunoglobulin; *MTX*, methotrexate; *WBC*, white blood cell.

**Fig 2:**
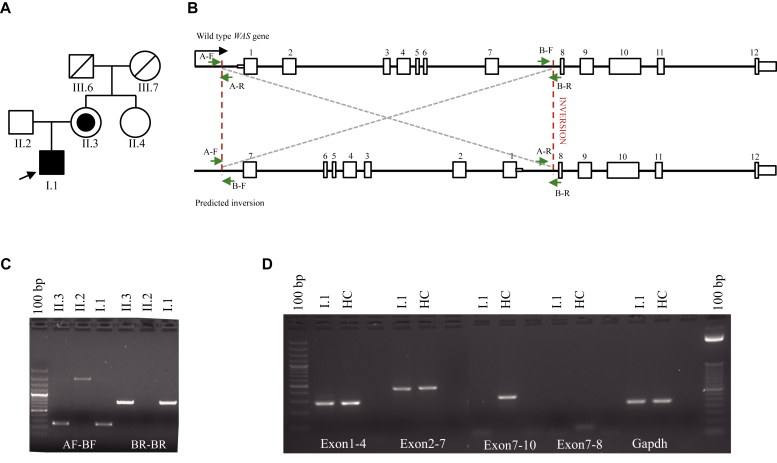
Identification of inversion in the *WAS* gene. **A,** Pedigree of the family. Proband is indicated by arrow. **B,** Graphical representation of predicted effects of inversion in the *WAS* gene. Primer design in the sites of inversion. **C,** DNA amplification with primers AF/BF and AR/BR in the family. Aspecific band in sample II.2. **D,** cDNA amplification with indicated primers.
